# LABAMPsGCN: A framework for identifying lactic acid bacteria antimicrobial peptides based on graph convolutional neural network

**DOI:** 10.3389/fgene.2022.1062576

**Published:** 2022-11-03

**Authors:** Tong-Jie Sun, He-Long Bu, Xin Yan, Zhi-Hong Sun, Mu-Su Zha, Gai-Fang Dong

**Affiliations:** ^1^ College of Computer and Information Engineering, Inner Mongolia Agricultural University, Hohhot, China; ^2^ College of Food Science and Engineering, Inner Mongolia Agricultural University, Hohhot, China

**Keywords:** lactic acid bacteria antimicrobial peptides, word embedding, tripeptide, graph convolution neural network, deep learning

## Abstract

Lactic acid bacteria antimicrobial peptides (LABAMPs) are a class of active polypeptide produced during the metabolic process of lactic acid bacteria, which can inhibit or kill pathogenic bacteria or spoilage bacteria in food. LABAMPs have broad application in important practical fields closely related to human beings, such as food production, efficient agricultural planting, and so on. However, screening for antimicrobial peptides by biological experiment researchers is time-consuming and laborious. Therefore, it is urgent to develop a model to predict LABAMPs. In this work, we design a graph convolutional neural network framework for identifying of LABAMPs. We build heterogeneous graph based on amino acids, tripeptide and their relationships and learn weights of a graph convolutional network (GCN). Our GCN iteratively completes the learning of embedded words and sequence weights in the graph under the supervision of inputting sequence labels. We applied 10-fold cross-validation experiment to two training datasets and acquired accuracy of 0.9163 and 0.9379 respectively. They are higher that of other machine learning and GNN algorithms. In an independent test dataset, accuracy of two datasets is 0.9130 and 0.9291, which are 1.08% and 1.57% higher than the best methods of other online webservers.

## 1 Introduction

Lactic acid bacteria (LAB) is a kind of bacteria that can use fermentable carbohydrates to produce large amounts of lactic acid (Gu et al., 2022; Hu et al., 2022). Organic acids, special enzymes, lactobacilli and other substances produced by lactic acid bacteria through fermentation have special physiological functions. A large number of research data show that lactic acid bacteria can promote animal growth, regulate the normal flora of gastrointestinal tract, maintain micro ecological balance, thereby improving gastrointestinal function, improving food digestibility and biological titer, reducing serum cholesterol, controlling endotoxin, inhibiting the growth of intestinal putrefactive bacteria, and improving the immunity of the body (Teusink and Molenaar, 2017). Lactic acid bacteria have been widely used in food industry and poultry husbandry, and also have important academic value in genetic engineering (Greub et al., 2016), biochemistry (Kadomatsu, 2022), genetics (Sung Won et al., 2020) and molecular biology (Saibil, 2022).

Antimicrobial peptides of lactic acid bacteria are a kind of active peptides or proteins produced by the metabolic process of lactic acid bacteria, which can inhibit or kill pathogenic bacteria or spoilage bacteria in food. In recent years, several new methods have been developed for the screening and development of new antimicrobial peptides, including enzyme-linked immunodeficient assay (Huang X et al., 2022), biological analysis of K+ ion current (Lauger and Apell, 1988), ATP-bioluminescence method (Crouch et al., 1993; Aiken et al., 2011), Lux gene-bioluminescence method (Van Dyk et al., 1994), berberine-based fluorescence analysis method (Liu et al., 1998; Song et al., 2018) and micro-plate method (Kai et al., 2012). Although the above wet experimental methods can distinguish, they are time-consuming and expensive, so they cannot be popularized and used. To help wet lab researchers identify novel antimicrobial peptides, a variety of computational methods for antimicrobial peptide identification have been proposed. Many algorithms combine machine learning or statistical analysis techniques such as discriminant analysis (DA) (Kouw and Loog, 2021; Beck and Sharon, 2022), fuzzy K-nearest neighbors (Zhai et al., 2020), hidden Markov models (Fuentes-Beals et al., 2022), logistic regression (Fagerland and Hosmer, 2012), random forests (RF) (Ziegler and Koenig, 2014), and support vector machines (SVM) (Azar and El-Said, 2014). Although these models have made great progress in antimicrobial peptide recognition, the following challenges still exist: First, many related classification tasks based on machine learning suffer from the small number of samples. The model trained with small sample size cannot achieve robustness and is prone to the problems of over fitting and poor generalization ability. Secondly, most of the existing feature extraction technologies are aimed at specific datasets, and do not have universality.

In a word, most of the existing machine learning based classification work mainly uses the manually determined features (Jiang et al., 2021), which is highly dependent on biologists. The artificially determined features also have their shortcomings. On the one hand, the intrinsic nonlinear information of the function of some peptides cannot be obtained through this featured way; On the other hand, when the research object is changed, the adaptability of artificial features is poor. In addition, the dimension disaster caused by feature engineering brings new troubles to researchers.

In the past 10 years, deep learning has achieved extremely rapid development. In the field of text processing, achievements in the application of natural language processing to biological information prediction have been published repeatedly. In particular, graph neural network plays an excellent role in text classification (Xie et al., 2022; Zhou et al., 2022). Qu (Qu et al., 2017) proposed a method based on deep learning to identify DNA binding protein sequences. This method uses a two-stage convolutional network to detect the functional domain of protein sequences, and uses LSTM neural networks to identify context dependencies. In the independent test set, the accuracy of the model in the yeast data set is 80%; Hamid and Friedberg (Hamid and Friedberg, 2018) proposed a method used word embedding and RNN to identify bacteriocin and non bacteriocin sequences. The recall of the model in the two training data sets is 89.8% and 92.1% respectively; Veltri (Veltri et al., 2018) proposed a deep neural network model, which includes an embedded layer, a convolution layer and a recursive layer. The accuracy of the model in the independent test set is 91.01%; Zeng (Zeng et al., 2019) proposed to identify protein sequences based on the use of node2vec technology, convolution neural network and sampling technology. In this framework, node2vec technology is used to capture the semantic features and topology of each protein in the protein interaction network, and convolution layer is used to extract information from gene expression profiles. The AUC of the model in the training set is 82%; he (He et al., 2021) proposed a new Meta learning framework based on mutual information maximization. The core of the framework is ProtoNet, a classical meta learning algorithm based on metric learning, which learns the vector representation of each prototype. The accuracy of this model in the training set of antifungal peptides was 91.3%. The above five deep learning models have improved the performance of AMP prediction to a certain extent, but most of these models used convolutional neural network and LSTM neural network combination framework without significant innovation. Recently, due to the rise of graph neural networks, more and more people began to do some research on graph neural networks. Therefore, our work is based on graph convolution neural network to predict LABAMPs.

In this work, we design a graph convolution neural network framework to predict antimicrobial peptides of lactic acid bacteria. First, we construct a large heterogeneous graph based on all the samples, which contains sequences and peptides (amino acids, dipeptide, tripeptide. We can think of these peptides as words in natural language processing) as nodes. Then connect the nodes by doing that: The edge between two peptide fragments is determined by whether the two peptide fragments appear together in the fixed range (window size) of a sequence. The edge between a peptide fragment and a sequence depends on whether the peptide fragment is a substring of this sequence. Finally, the classification of nodes on the graph is realized through the calculation and transmission of information between nodes on the graph. The experimental results show that our model has great advantages over machine learning methods, deep learning models and other webservers.

## 2 Materials and methods

### 2.1 Collection of datasets

We collected LABAMPs records from 25 databases (Gueguen et al., 2006; Mulvenna et al., 2006; Fjell et al., 2007; Henderson et al., 2007; Kawashima et al., 2008; Hammami et al., 2009; Hammami et al., 2010; Sundararajan et al., 2012; Gogoladze et al., 2014; Theolier et al., 2014; Pirtskhalava et al., 2021; Shi et al., 2022) according to the 30 genus classification of lactic acid bacteria in Supplementary Table S1. Finally, after removing duplicate records, 1622 LABAMPs are obtained, and their lengths are from 2 to 1619.

According to the positive raw data set obtained above, we do some processing on it: First, we remove records which contain unnatural amino acids such as B, J, O, U, X, and Z from these raw data. Second, to reduce sequence homology bias and redundancy, we used respectively the CD-HIT program (Li and Godzik, 2006) to delete peptides with 70% and 90% similarity to each other. Finally, we get 460 and 636 peptide sequences after removing redundancy, respectively.

Our negative raw datasets obtained as follows:

1 On the UniProt website (Consortium, 2021), we obtain peptide sequences between the length of 2–1619;
2 Remove sequences contain or annotated with information of antimicrobial, antibiotic, fungicide, defensin, AMP, membrane, toxic, secretory, defensive, anticancer, antiviral, antifungal, effector, and exacted;
3 Remove resulting protein sequences which include unnatural amino acids;
4 Remove peptide sequences with 70% and 90% similarity by CD-HIT program;
5 Randomly select the same number of sequences as the number of positive samples.

All positive and negative samples are shown as Table 1, with processing of 70% and 90% by CD-HIT. We called them DS-70% and DS-90% respectively. The statistics of the preprocessed datasets are summarized in Table 2. Since we classify nodes on the graph, the number of graphs is one respectively in DS-70% and DS-90%. The number of sequences is the total number of positive and negative samples of DS-70% or DS-90%. The number of words is obtained by removing stop words and the words whose frequencies are less than 5. The number of nodes is the sum of the number of sequences and the number of words. Because our work has two categories of tasks, the number of categories is two.

**TABLE 1:** Raw data processed through CD-HIT program.

Attribute	Raw data	DS-70%	DS-90%
AMPs	1622	460	636
nonAMPs	1622	460	636

**TABLE 2:** Summary statistics results of datasets.

Attributes	Datasets	
	DS-70%	DS-90%
Graphs	1	1
Sequences	920	1272
Words	7455	7621
Nodes	8375	8893
Classes	2	2

### 2.2 Model construction

The model construction is divided into three steps: first, establish the initial graph, then conduct the convolution operation on the graph, and finally complete the node classification through the classification function.

#### 2.2.1 Establishment of initial graph

Before the construction of initial graph, we preprocess all positive and negative samples. First, all positive and negative samples are segmented by amino acid, dipeptide or tripeptide as words. Secondly, count the words frequencies, and filter all the words whose word frequency is less than 5 times. Then, we get the required words.

**FIGURE 1:**
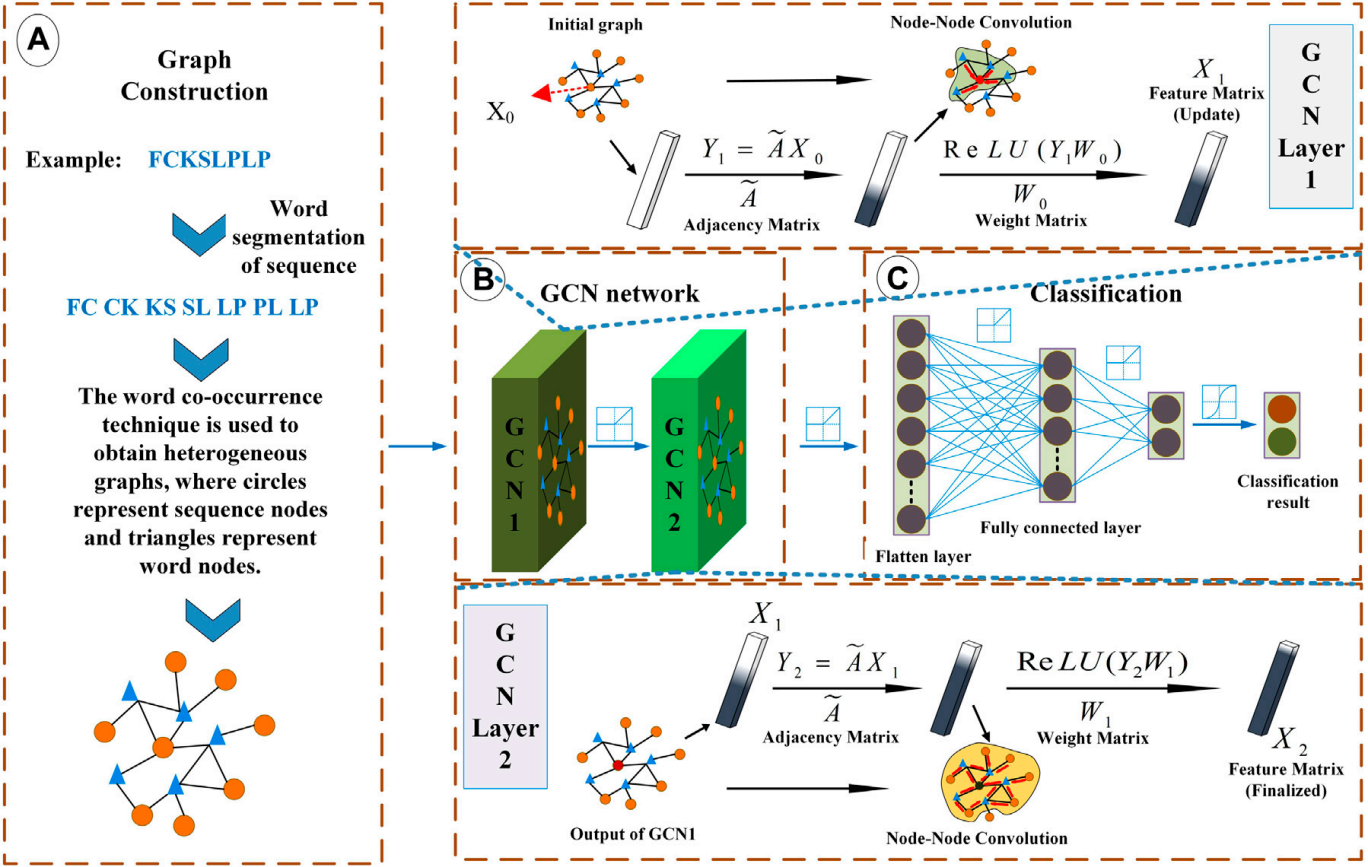
Overview of LABAMPsGCN model architecture. **(A)** Graph Construction. Each sequence is processed by word segmentation, and then the required graph is obtained by word co-occurrence technology. **(B)** Graph convolutional neural network. It mainly carries out message transmission through word-sequence relations. **(C)** Classification. It uses the full connection layer for classification.

Suppose the initial input graph is expressed as Graph G=(V,E) , then the number of V is equal to the sum of the number of sequences and the number of peptide segments, and the number of edges depends on the connecting lines between peptide segments and the connecting lines between peptide segments and sequences. As shown in Figure 1A, there are two kinds of edges. One kind of edges are the connecting lines between peptide segments—if two peptide segments occur at the same time within the specified range of the same sequence, the corresponding nodes of these two peptide segments will be connected. The other kind of edges are the connection lines between peptide segments and sequences—if a peptide segment is a sub string of a sequence, the corresponding nodes will be connected.

In order to calculate the information on the graph through the edges, we establish the adjacency matrix *A* of the initial graph, that is, assign a certain weight to each edge. The calculation method is shown in Eq. 1. Where |W| represents the total number of sliding windows in all sequences, and its value is a positive integer. |W(i)| is the number of sliding windows containing peptide segment i in all sequences, and |W(i,j)| is the number of sliding windows containing both peptide segment i and peptide segment j in all sequences. $n_{i,j}$ is the number of occurrences of the peptide segment i in sequence *j*, and *N* is the total peptide number of all sequences. |D| is the total number of all sequences, and |{j:i∈j}| is the number of sequences containing peptide segment i . The reason for adding one to the denominator is that when the peptide segment is not in all known sequences, |{j:i∈j}| will be zero. Therefore, one is added to denominator.

$$A_{ij}=\left\{ \begin{array}{cc} \log\frac{\left|W\right|\cdot \left|W\left(i,j\right)\right|}{\left|W\left(i\right)\right|\cdot \left|W\left(j\right)\right|} & i,j\;are\;words\\ \frac{n_{i,j}}{N}\cdot \log\frac{\left|D\right|}{\left|\left\{j:i\in j\right\}\right|+1} & i\;is\;word,\;j\;is\;sequence\\ 1 & i=j\\ 0 & otherwise \end{array}\right.$$ (1)

#### 2.2.2 Graph convolutional network module

Word embedding is a method converting a word into a vector representation. There are many methods for word embedding, such as one-hot embedding, Skip Gram model (Carrasco and Sicilia, 2018), CBOW model (Xiong et al., 2019) and GloVe word vector (Gao and Huang, 2021). In this module, we first need to determine the node features of the initial graph. We use one-hot embedding to embed each word and send it to the model together with the sequence for training. Because the initial values of node features have little influence on the graph convolution neural network, we set X as the identity matrix I .

Since the diagonal element of the adjacency matrix is 0, it is easy to lose the information of the nodes themselves in the calculation process, so an identity matrix is added to the adjacency matrix. In order to avoid the change of feature distribution, the adjacent matrix with an identity matrix is normalized to obtain the processed adjacent matrix Norm(A+I) (Gao and Huang, 2021).

We design a graph convolution neural network framework to learn the information between nodes on the graph and transfer the related information under the supervision of labels, and finally achieve node classification. The graph convolution neural network framework of lactobacillus antibacterial peptides can be expressed as Eq. 2.

$$R=soft\max\left(Norm\left(A+I\right)...ReLU\left(Norm\left(A+I\right)XW_{0}\right)...W_{n}\right)$$ (2)

The network learning process under the supervision of sequence labels needs to calculate the loss rate, and we use the cross entropy loss function to calculate the loss (Aurelio et al., 2019). Eq. 2 is a general model of LABAMPsGCN. Figure 1B shows a two-layer LABAMPsGCN. In the following chapters, we analyze that the two-layer LABAMPsGCN has the best performance.

#### 2.2.3 Classification module

We use the full connection layer to integrate the feature space into the sample label space, and then use the *softmax* classification function to calculate the probability of nodes being classified into different categories. As is shown in Figure 1C.

### 2.3 Evaluation metrics

To assess the performance of LABAMPsGCN, we adopt statistical metric of precision, recall, accuracy and F1_score . They defined as follows:

$$\mathrm{Pr}ecision=\frac{TP}{TP+FP}$$ (3)

$$Recall=\frac{TP}{TP+FN}$$ (4)

$$Accuracy=\frac{TP+TN}{TP+TN+FP+FN}$$ (5)

$$F1\_score=2\times \frac{\mathrm{Pr}ecision\times Recall}{\mathrm{Pr}ecision+Recall}$$ (6)

TP , TN , FP and FN are the four components of the confusion matrix, and also are the abbreviation of true positive, true negative, false positive and false negative, respectively. Precision rate means the proportion of correctly predicted positive to all actually positive samples. Recall rate means the proportion of correctly predicted positive samples to all the samples that should be predicted to be positive samples. Accuracy means the percentage of correct predictions in all samples. F1_score denotes the harmonic value of precision and recall.

### 2.4 Implementation details

The parameters of a model have an important impact on the performance of the model. In our LABAMPsGCN, we set the activation function, window size, first layer convolution size, learning rate and loss rate to ReLU, 15, 200, 0.01, and 0.5 respectively. We used Adam optimizer (Shao et al., 2021) to train our model for 150 epochs.

### 2.5 Development of the webserver.

We constructed a webserver with our prediction model embedded at the back end of website. When users submit their interested LABAMPs, the predicted percentage will be displayed based on the website prediction tool (Sim et al., 2012). Because the weight matrix of the graph convolution neural network will change with the change of the adjacency matrix and feature matrix of the input data, we embedded SVM model with accuracy lower of 3.77% than that of LABAMPsGCN.

## 3 Results

### 3.1 Effects of different feature extraction methods on graph convolutional neural networks

We randomly combined the features of single peptide, dipeptide and tripeptide respectively, and obtained six feature combinations: dipeptide, dipeptide and single peptide, tripeptide, tripeptide and single peptide, tripeptide and dipeptide, tripeptide added by dipeptide and single peptide. Table 3 shows the model accuracy on the DS-70% and DS-90%.

**TABLE 3:** The different accuracy of different features on LABAMPsGCN.

Features	Number of features	Datasets	
		DS-70%	DS-90%
D^a^	400	0.8913	0.9088
D + S^b^	420	0.8870	0.9010
T ^c^	8000	0.9098	0.9340
T + S^d^	**8020**	**0.9163**	**0.9379**
T + D^e^	8400	0.9076	0.9277
T + S + D^f^	8420	0.9065	0.9285

^a^ D: Dipeptide.

^b^ D + S: Dipeptide + Single peptide.

^c^ T: Tripetide.

^d^ T + S: Tripetide + Single peptide.

^e^ T + D: Tripetide + Dipetide.

^f^ T + S + D: Tripetide + Single peptide + Dipetide.

Note: the bold value in table means the best value.

It can be seen that the features of tripeptide and single peptide are significantly better than other combinations on DS-70% and DS-90%. As the number of features continues to increase, the accuracy (ACC) of the test data is also slowly increasing, and the number of features in DS-70% and DS-90% begins to decline significantly after 8020.

### 3.2 Parameter sensitivity

#### 3.2.1 Window sizes

**FIGURE 2:**
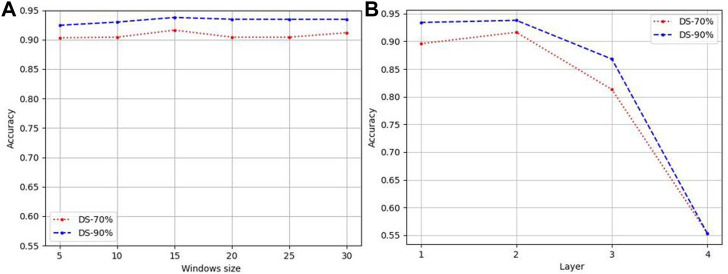
Parameter analysis of LABAMPsGCN. **(A)** Accuracy varied by windows size. **(B)** Accuracy varied by numbers of layers.

Figure 2A reports the accuracy for different sliding window on DS-70% and DS-90% based on features of tripeptide and single peptides. It demonstrates that the influence of the size of the sliding window on the prediction accuracy meets the general rule—taking 15 as the dividing point, it rises first and then falls. It is further explained that small windows cannot accommodate sequence fragments that play key functions, while too large windows take some irrelevant information as key information to participate, disturbing the judgment. Therefore, in this paper, window size is set to 15.

#### 3.2.2 Graph convolutional network layer

We designed GCNs with different layers to obtain the features of LABAMPs. Figure 2B indicate the effect of the number of GCN layers on the performance of our model. In this paper, we changed the GCN layer in {1, 2, 3, 4}. It can be seen from Figure 2B that the 2-layer GCN can achieve the optimal performance. Too many GCN layers will cause the model to be over-smoothing, thus causing the learned model to collapse. Although there is no direct sequence-sequence edge connection in the graph, 2 GCNs can be connected through the middle word node, thus realizing sequence to sequence information interaction.

If there are too many layers, the features of a node will aggregate the features of more and more neighbors, so that these nodes become similar, which increases the similarity between classes, and the natural classification effect is poor.

### 3.3 Compare with machine learning methods

In order to verify metric of LABAMPsGCN, we compare machine learning models with it on the same features. In Table 4 all results are obtained by using 10-fold cross-validation. We used Multinomial Bayesian classifier (MNB), Random forest (RF), Support vector machine (SVM), AdaBoost (Huang H et al., 2022) and XGBoost (Zhang et al., 2022). It can be seen that LABAMPsGCN show good performance no matter how features change. This is because LABAMPsGCN can obtain the information of sequence nodes through word nodes.

**TABLE 4:** Comparisons of LABAMPsGCN with machine learning and GNN models.

Datasets	Models	Features					
		D^a^	D + S^b^	T^c^	T + S^d^	T + D^e^	T + S + D^f^
DS-70%	MNB^g^	0.8457	0.8283	0.8391	0.8391	0.8391	0.8391
	RF^h^	0.8446	0.8576	0.7989	0.7891	0.7957	0.7978
	SVM^i^	0.8554	0.8663	0.8402	0.8402	0.8402	0.8402
	AdaBoost	0.7946	0.8196	0.7348	0.7348	0.7348	0.7348
	XGBoost	0.8489	0.8685	0.7793	0.7793	0.7793	0.7793
	GNN^j^	0.8596	0.8549	0.8836	0.8916	0.8513	0.8499
	LABAMPsGCN	**0.8913**	**0.8870**	**0.9098**	**0.9163**	**0.9076**	**0.9065**
DS-90%	MNB	0.8586	0.8461	0.8585	0.8585	0.8585	0.8585
	RF	0.8776	0.8576	0.8383	0.8218	0.8359	0.8281
	SVM	0.8800	0.8842	0.9002	0.9002	0.8988	0.8987
	AdaBoost	0.8334	0.8328	0.7558	0.7558	0.7558	0.7558
	XGBoost	0.8776	0.8791	0.8131	0.8131	0.8131	0.8131
	GNN	0.8810	0.8897	0.9019	0.9146	0.8946	0.8943
	LABAMPsGCN	**0.9088**	**0.9010**	**0.9340**	**0.9379**	**0.9277**	**0.9285**

^a^ D: Dipeptide.

^b^ D + S: Dipeptide + Single peptide.

^c^ T: Tripetide.

^d^ T + S: Tripetide + Single peptide.

^e^ T + D: Tripetide + Dipetide.

^f^ T + S + D: Tripetide + Single peptide + Dipetide.

^g^ MNB: Multinomial naïve Bayes.

^h^ RF: Random Forest.

^i^ SVM: Support Vector Machine.

^j^ GNN: graph neural network.

Note: the bold value in table means the best value.

### 3.4 Comparison with existing AMP prediction tools

Table 5 compares our LABAMPsGCN model to three state-of-the-art machine learning methods which can be found publicly for AMPs recognition. Table 5 shows that our LABAMPsGCN model achieves the best values of metrics for Recall, Precision and Accuracy. In DS-70%, the Recall score of AMPfun model (Chung et al., 2020) is the highest (3.42% higher than our model). In DS-90%, the metrics of our LABAMPsGCN model are significantly better than other methods.

**TABLE 5:** Comparisons of LABAMPsGCN with three state-of-the-art webservers.

Datasets	Tool	R^a^	P^b^	ACC^c^
DS-70%	CAMP-SVM	0.8696	0.8889	0.8804
	iAMP-2L	0.875	0.9333	0.9022
	AMPfun	**0.8913**	0.9111	0.9022
	LABAMPsGCN	0.8571	**0.9556**	**0.9130**
DS-90%	CAMP-SVM	0.8852	0.871	0.8819
	iAMP-2L	0.8889	0.9032	0.8976
	AMPfun	0.8923	0.9355	0.9134
	LABAMPsGCN	**0.9077**	**0.9516**	**0.9291**

^a^ R: Recall.

^b^ P: Precision.

^c^ ACC: accuracy.

Note: the bold value in table means the best value.

### 3.5 Ablation study

In order to judge if all the parts of our identifier are necessary, we adopt three variants of LABAMPsGCN (LABAMPsGCN-noFC, LABAMPsGCN-cheby and LABAMPsGCN-cheby-noFC) as comparison methods. Specifically, LABAMPsGCN-noFC means that we do not add a full connection layer after the GCN layer for classification, while directly use the output of the GCN layer for classifying. LABAMPsGCN-cheby adds Chebyshev polynomials (Christiansen et al., 2021), which can use polynomial expansion to approximate the convolution of graphs, that is, polynomial approximation of parameterized frequency response functions. LABAMPsGCN-cheby-noFC adds Chebyshev polynomials and there is no full connection layer after GCN layer output.

Table 6 shows the evaluation indicators obtained by training with LABAMPsGCN and its variants on DS-90%. These four groups of training were conducted on the feature of tripeptide and single peptide. For LABAMPsGCN and LABAMPsGCN-noFC, the ACC of LABAMPsGCN was significantly higher than that of LABAMPsGCN-noFC. This is because the full connection layer integrates the feature representations and maps them to the space where the sample labels are located. For LABAMPsGCN and LABAMPsGCN-cheby, the performance of LABAMPsGCN-cheby is slightly poor because the use of Chebyshev polynomials makes each sequence vertex fuse too much irrelevant information. For LABAMPsGCN and LABAMPsGCN-cheby-noFC, the performance of LABAMPsGCN with full connection layer is significantly higher than that without it.

**TABLE 6:** Performance evaluation of LABAMPsGCN and its three variants.

Methods	R^a^	P^b^	ACC^c^	F1-score
LABAMPsGCN	**0.9492**	**0.9032**	**0.9291**	**0.9256**
LABAMPsGCN-noFC	0.8906	0.9194	0.9055	0.9048
LABAMPsGCN-cheby	0.803	0.8413	0.8189	0.8217
LABAMPsGCN-cheby-noFC	0.7846	0.8095	0.7874	0.7969

^a^ R: Recall.

^b^ P: Precision.

^c^ ACC: accuracy.

Note: the bold value in table means the best value.

### 3.6 Visualization of words

**FIGURE 3:**
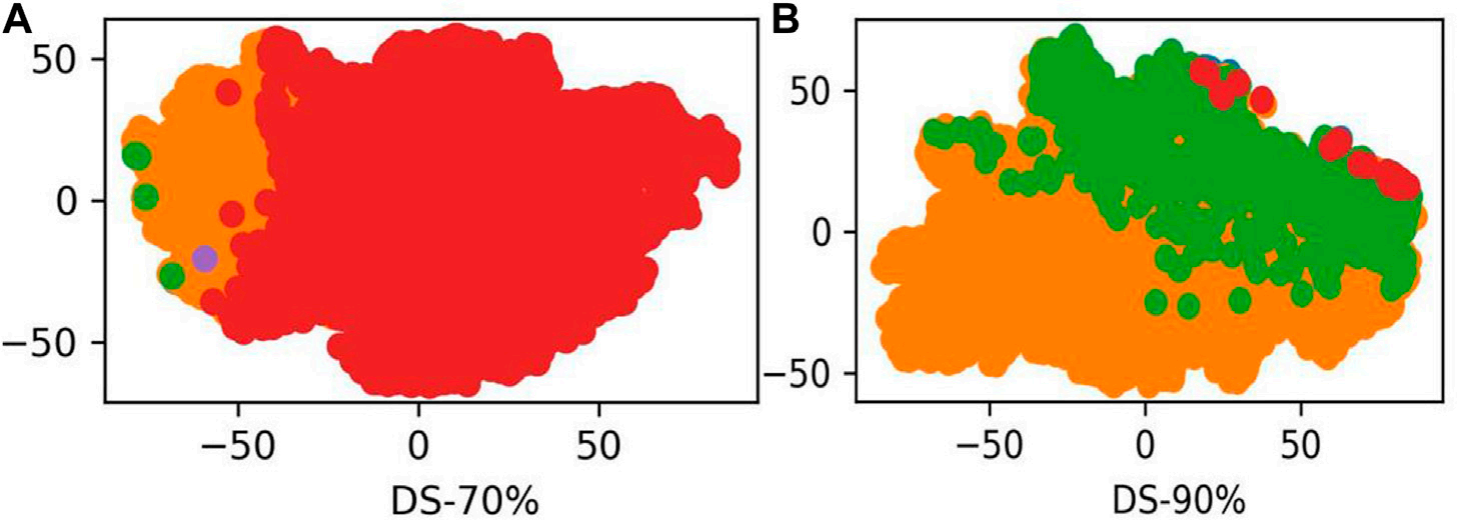
The t-SNE visualization of the second layer features learned from DS-70% and DS-90%. **(A)** The second word features learned from DS-70%. **(B)** The second word features learned from DS-90%.

LABAMPsGCN learned a lot of word features related to labels. In order to observe these words clearly, we visualized them qualitatively. Figure 3 shows the t-SNE visualization (Ruit et al., 2022) of the second layer word features learned from DS-70% and DS-90%. We set the dimension of the maximum value in the word feature vectors as the label of the word. As can be seen from Figure 3, words of the same color are clustered together, which means that a large number of words are closely related to certain specific classes. The red, green and orange in Figure 3 are used for visualization to determine whether word embedding can learn the main information of some sequences. Different colors in the figure represent different sequences. Figure 3A and Figure 3B is the results of DS-70% and DS-90%, respectively. In Table 7, we show the top representative words in each category, such as “ILE,” “TIW,” and “KLK”.

**TABLE 7:** Words with highest ACCs for two datasets of DS-70% and DS-90%. We used the word embedding at the last level of GCN to view the best performing words under each category.

DS-70%		DS-90%	
LABAMPs	nonLABAMPs	LABAMPs	nonLABAMPs
ILE	YET	GSG	FAD
TIW	MAV	CIV	EAE
KLK	RNF	KYR	GHH
KDF	LCH	SAV	KPP
GDH	RSS	WHT	FKF
YQN	WAL	NAV	FIL
GTW	FGW	IQS	VMM
MPI	WSG	EYE	PTD

## 4 Discussion

In this study, we constructed LABAMPsGCN, a novel graph-based identifier to predict LABAMPs accurately. In this identifier, we designed a graph convolutional neural network framework to automatically learning sequence features. By retrieving and reorganizing multiple AMPs databases and Uni-Prot database, we constructed the positive and negative datasets. We organized positive and negative samples into a large heterogeneous graph, transforming the sequence classification problem into a node classification problem. Graph convolution neural network can aggregate the information of the surrounding nodes to predict the label information of the central node.

LABAMPsGCN is superior to other predictors, on the one hand, because the graph structure can effectively represent the relationship between sequences and words (when constructing a graph, an edge is established between a word and a sequence when this word belongs to this sequence), on the other hand, the label information of sequences can be transferred through the edges on the graph. Because the graph structure is a kind of many-to-many structure, the label information of sequences can be transferred in the whole graph. In this way, the words corresponding to positive and negative labels can be easily distinguished. These words may be the key features to determine whether a sequence is a LABAMP.

For users’ convenience, we have established a publicly accessible web server (http://www.dong-group.cn/database/dlabamp/Prediction/amplab/result/) that can help to predict LABAMPs metabolized from various Lactic acid bacteria. In the next step, we will discuss how to mine the key fragments with antimicrobial function from the whole genome sequence by combining information such as multiple sequence alignment and domain prediction. We believe LABAMPsGCN will be a competent tool for screening lactic acid strains with antimicrobial activities.

## Data Availability

The datasets presented in this study can be found in online repositories. The names of the repository/repositories and accession number(s) can be found in the article/Supplementary Material.
