# Supplementary material for: LABAMPsGCN: A framework for identifying lactic acid bacteria antimicrobial peptides based on graph convolutional neural network
Source: Front Genet. 2022 Nov 3;13:1062576. doi: 10.3389/fgene.2022.1062576 (PMC9669054; doi:10.3389/fgene.2022.1062576)
Supplement: Supplementary file 1 [file DataSheet1.PDF]

Supplementary Material

1 Supplementary Tables

Table 1: Species and genus of lactic acid bacteria. We collected LABAMPs records from 25 databases according to the 30 genus classification of lactic acid bacteria.

Number	bacteria category
1	Fervidobacterium
2	Thermotoga
3	Lachnospira
4	Ruminococcus
5	Mitsuokella
6	Thermoanaerobic
7	Halobacterium
8	Bacillus
9	Exiguobacterium
10	Staphylococcus
11	Gemella
12	Lactobacillus
13	Paralactobacillu
14	Aerococcus
15	Pediococcus
16	Carnobacterium
17	Isobaculum
18	Marinilactobacillus
19	Enterococcus
20	Atopobium
21	Melissococcus
22	Tetragenococcus
23	Vagococcus
24	Leuconostoc
25	Oenococcus
26	Weissella
27	Streptococcus
28	Lactococcus
29	Abiotrophia
30	Alloiococcus
